# Tumor in the Cerebellum of a Child

**Published:** 1822-04

**Authors:** A. C. Hutchison

**Affiliations:** Surgeon Extraordinary to H. R. H. the Duke of Clarence, &c.


ART. IV.—*Tumor in the Cerebellum of a Child.* By the same.

MRS. SIMS, of No. 15, Stacey-street, St. Giles', applied at the Royal Infirmary for Sick Children, in July last, to solicit advice for her child, a boy, between nine and ten years old, who was labouring under chronic hydrocephalus; and who had been totally blind from the first attack of the disease, which was some months previous to this period.

The patient came under the care of Dr. GRANVILLE, who prescribed for him; but the symptoms continuing, after the lapse of a few weeks, without any alleviation, I suggested to him to try the effects of pressure; as I had, at the request of Sir GILBERT BLANE, bandaged the heads of two children, under similar circumstances, and which were then in progress of recovery.*

Due attention being paid to keep the child's bowels freely open in the mean while, this plan was persisted in to the period of his decease, which occurred yesterday, (the 19th of January;) and it is highly worthy of remark, that the child always cried, and complained much of pain in the head, when the bandage happened either to be loose or removed for re-application.

The child's head was this day examined by me, in the presence of Dr. GRANVILLE and Mr. M. DALZELL; and the appearances on dissection were the following:

The head much enlarged, and the sutures all in contact, without the smallest appearance, any where, of separation, as is

* Sir G. Blane has since published a paper on this subject, in the *London Medical and Physical Journal*, for October 1821.

usual in such cases; and the os frontis bore no marks of ever having been two distinct bones. The cranium was thinner than natural in an individual of this age; and the calvarium was very readily removed; the adhesions between it and the dura mater being but slight. The dura mater was found natural; but the vessels of the pia mater were injected with blood, and turgid.

On slicing off the brain from each hemisphere, we laid open the lateral ventricles about a quarter of an inch, only, below the surface; both which were very much distended with a clear fluid, amounting to about a pint and a half. The lining membrane of the ventricles was exceedingly tough, and traversed with highly-injected blood-vessels. Nothing else extraordinary was observed in the brain or nerves, nor did any thing remarkable appear on viewing the cerebellum *in situ*; but, when the medulla oblongata was divided, and the cerebellum removed, we found a tumor the size of a very small orange, of a schirrous hardness, and situated in the posterior and inferior part of the os occipitis: when this tumor was cut into, it had precisely the appearance of cow's udder. There was no mark of external injury ever having been inflicted over the situation of the tumor; neither did the father or mother ever recollect the child having fallen, so as to fracture, or otherwise injure, this part of the head.

It appears to me that there are some interesting facts in the history of this case, well worthy the attention of the profession; more particularly the striking advantage that seemed to be derived from pressure by bandage in chronic hydrocephalus, as recommended by Sir Gilbert Blane.

*Leicester-square; 20th January*, 1822.

